# Prospective cholestanol screening of cerebrotendinous xanthomatosis among patients with juvenile-onset unexplained bilateral cataracts

**DOI:** 10.1186/s13023-022-02591-4

**Published:** 2022-12-13

**Authors:** Gorka Fernández-Eulate, Gilles C. Martin, Pascal Dureau, Claude Speeg-Spatz, Anais Brassier, Perrine Gillard, Dominique Bremond-Gignac, Dominique Thouvenin, Cecile Pagan, Foudil Lamari, Yann Nadjar

**Affiliations:** 1 Reference Center for Lysosomal Diseases, Neuro-Metabolism Unit, Neurology Department, Pitié-Salpêtrière University Hospital, APHP, 47-83 Boulevard de l’Hôpital, 75013 Paris, France; 2 Reference Center for Neuromuscular Diseases, Neuro-Myology Department, Pitié-Salpêtrière University Hospital, APHP, Paris, France; 3 Ophthalmology Department, Rothschild Foundation Hospitals, Paris, France; 4 Ophthalmology Department, Nouvel Hôpital Civil - Strasbourg University Hospital, Strasbourg, France; 5 Pediatric Department, Necker Enfants Malades University Hospital, APHP, Paris, France; 6 Ophthalmology Department, Necker Enfants Malades University Hospital, APHP, INSERM Unit 1138, Team 17, Cordeliers, Paris University, Paris, France; 7 Ophtalmologie Rive Gauche Clinic, Toulouse, France; 8 Department of Biochemistry and Molecular Biology, LBMMS, Lyon University Hospital, Lyon, France; 9 Metabolic Biochemistry Unit, Department of Biochemistry of Neurometabolic Diseases, Pitié-Salpêtrière University Hospital, Paris, France

**Keywords:** Cataract, Cerebrotendinous xanthomatosis, Cholestanol, Inborn errors of metabolism

## Abstract

**Background:**

Cerebrotendinous xanthomatosis (CTX) is a rare genetic disorder related to *CYP27A1* biallelic mutations, leading to decreased synthesis of bile acids and increased cholestanol. Juvenile bilateral cataracts are one of the most common findings in the disease, frequently occurring before the onset of neurological manifestations. While early treatment with chenodeoxycholic acid can prevent the onset of neurological impairment, poor awareness of CTX accounts for a markedly delayed diagnosis. The objective of this study was to evaluate the utility of plasma cholestanol analysis at the moment of cataract diagnosis and before the onset of neurological impairment in CTX.

**Methods:**

Multicenter prospective cohort study of patients with juvenile-onset unexplained bilateral cataracts recruited from seven French ophthalmology departments. Plasma cholestanol analysis was performed at diagnosis from January 2018 to January 2020. CYP27A1 genetic testing was performed at the ophthalmologist’s discretion. Cholestanol levels were compared with those of a similar population of patients without cataracts (control cohort).

**Results:**

30 patients were finally recruited, with a mean age at cataract diagnosis of 7.1 years (± 4.8 SD, range 1–19 years). One patient had a very high cholestanol level (68 μmol/L, reference < 10) and carried two pathogenic heterozygous mutations in *CYP27A1* confirming CTX. This patient was a 19-year-old female, reporting chronic diarrhea only in childhood, and diagnosed with bilateral posterior cataracts with cortical fleck-like opacities. Therefore, the incidence of CTX in our cohort of patients was 3.3%. Five further patients (5/29; 17.2%) had moderate elevations of cholestanol level (between 10.3 and 16.5 μmol/L), compared to 12/286 (4.2%) in the control cohort (*p* = 0.014) after adjustment for age.

**Conclusion:**

Our study argue for the relevance of plasma cholestanol CTX screening in all patients with juvenile-onset unexplained cataracts, even without other CTX identified manifestations. Whether moderate elevations of plasma cholestanol unrelated to CTX may be a risk factor for bilateral cataracts occurrence needs further examination.

## Background

Cerebrotendinous xanthomatosis (CTX) is a rare autosomal recessive disorder caused by sterol 27-hydroxylase deficiency (encoded by *CYP27A1*), leading to a decreased synthesis of bile acids with consecutive abnormal production of cholestanol. This increased cholestanol accumulates in different biological fluids and tissues, including the blood (making plasma cholestanol an easily obtainable and sensitive diagnostic biomarker), the central nervous system, and the lens [1]. Bilateral cataracts are present in more than 80% of CTX patients, develop early in the course of the disease [1], and can be associated in children with chronic diarrhea and/or neurodevelopmental disorders. Treatment with chenodeoxycholic acid (a bile acid) normalizes cholestanol levels and may prevent the onset or progression of neurological symptoms that usually occur in adulthood, leading to significant motor, cognitive and behavioral disability. However, this is mostly true if patients are treated early in the course of the disease [2, 3], notably at the moment of cataract diagnosis. Unfortunately, due to poor awareness of CTX, time from onset of symptoms to diagnosis can be up to 20–25 years[4]. Our objective was to evaluate for the first time the utility of prospective plasma cholestanol analysis for the diagnosis of CTX in patients presenting with unexplained juvenile-onset bilateral cataracts.

## Methods

From January 2018 to January 2020, seven French ophthalmology departments prospectively assessed plasma cholestanol levels in all patients with unexplained bilateral cataracts diagnosed between 1 and 20 years of age, as part of standard care. For recruited patients (the “cataract cohort”), in addition to cholestanol measurement, data was collected including family history of neurological diseases, personal history of learning and motor difficulties and diarrhea, and cataract morphology. Patients had undergone a negative toxoplasmosis, rubella, cytomegalovirus, herpes simplex and HIV (TORCH) screening.

Plasma cholestanol analysis was performed by quantitative liquid chromatography–mass spectrometry analysis at Pitié-Salpêtrière Hospital and Lyon Hospital’s laboratories (reference range < 10 µmol/L). Biochemists prompted ophthalmologists to perform *CYP27A1* genetic testing for patients with high cholestanol levels. Patients with moderate elevations of plasma cholestanol underwent an hepatic workup and the cholestanol µmol/L/cholesterol mmol/L ratio was ascertained (reference range 0.728–2.716).

In order to compare the cholestanol levels with those from a similar population of patients without cataracts, a “control cohort” was retrospectively generated from patients within the same range of age and period of time who had a plasma cholestanol analysis for other reasons than cataract (juvenile psychosis, intellectual disability and leukodystrophy).

Collected clinical data were summarized using descriptive statistics. A Chi-square and a one-tailed T-test were performed to compare the number of patients with elevated cholestanol levels and the mean cholestanol level respectively between the cataract and control groups. A logistic regression was performed to adjust the proportion of patients with elevated cholestanol to age.

Patients or legal representatives gave their written consent for the use of their anonymized data. This study was conducted according to French legislation and authorized by the National Commission on Informatics and Liberty (no. 2211991).

## Results

**Fig. 1:**
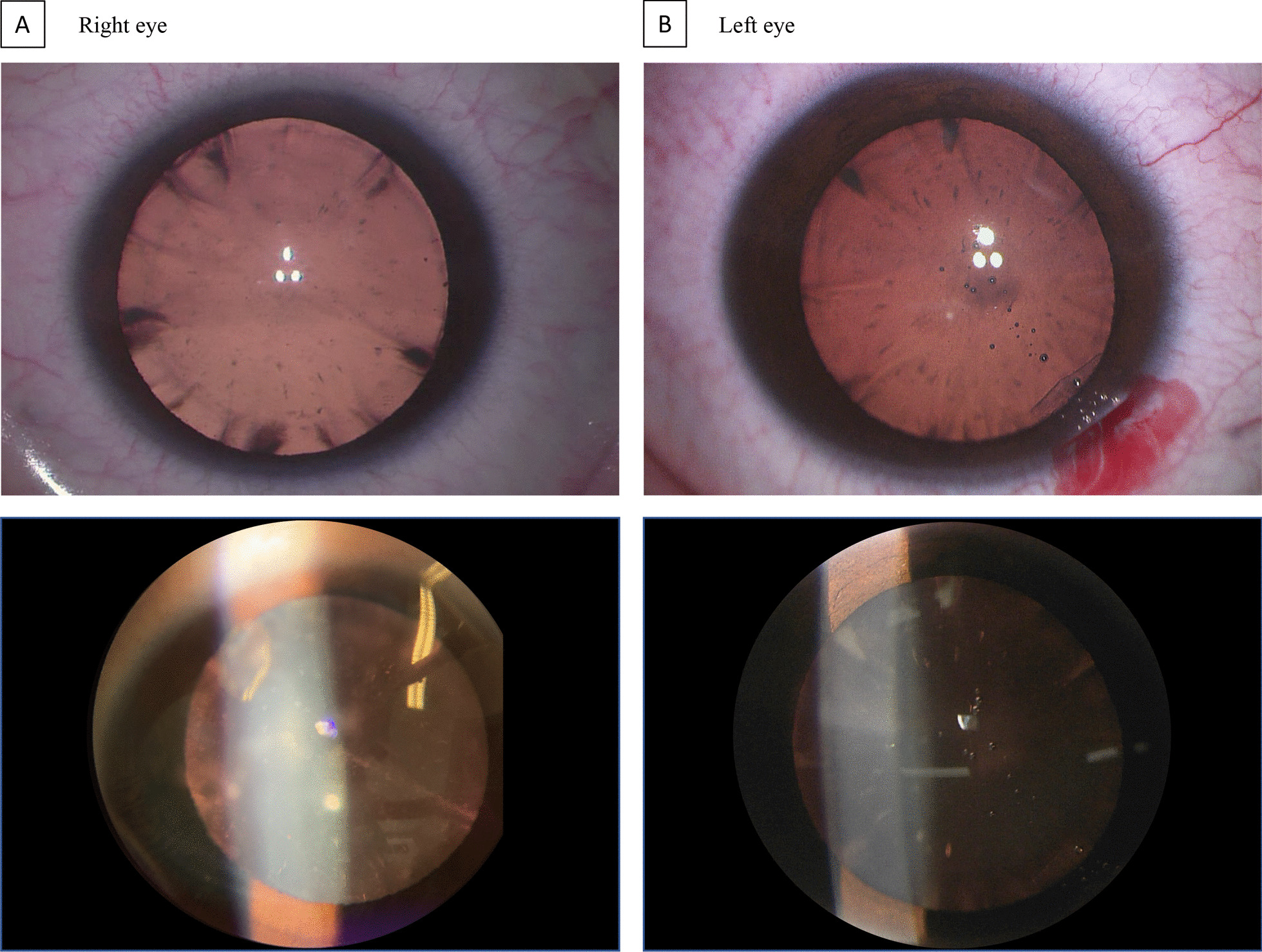
Posterior cataract with cortical “flecklike” opacities in the patient diagnosed with Cerebrotendinous Xanthomatosis

Thirty patients were enrolled and seventeen were females (56.7%), with a mean age at cataract diagnosis of 7.1 years (± 4.8 SD, range 1–19 years) (Table 1). Six patients had cholestanol levels higher than the upper reference range, with five considered moderate (between 10.3 and 16.5 µmol/L) and one very high (68 µmol/L). For this last 19-year-old female patient, two heterozygous mutations in *CYP27A1* confirmed CTX. She had a personal history of unexplained diarrhea in childhood, which improved during adolescence, and no neurodevelopmental disorder. She had bilateral posterior cataracts with cortical fleck-like opacities (Fig. 1). Upon neurological examination, only a fine postural tremor of the upper limbs was observed with no other clinical abnormality. Treatment with chenodeoxycholic acid was started, leading to a normalization of cholestanol levels at the 6 month visit.

**Table 1:** Demographic characteristics of patients with juvenile-onset bilateral cataracts

Demographic characteristics	Patients with juvenile-onset bilateral cataract (n = 30)
Female, n (%)	17 (56.7)
Age at inclusion, y, mean ± SD	7.1 ± 4.8
*Localization of cataract*	
Nuclear	11 (36.7)
Cortical	4 (13.3)
Cortico-nuclear	3 (10)
Anterior	2 (6.7)
Posterior	6 (20)
Total	4 (13.3)
Family history of neurological disease, n (%)	3 (10)
Consanguinity, n (%)	5 (16.7)
Learning difficulties, n (%)*	7 (24.1)
Motor difficulties, n (%)*	3 (10.4)
Diarrhea, n (%)**	2 (9.1)

*Y* years

*Out of 29 patients (1 missing data)

**Out of 22 patients (8 missing data)

Thus, the rate of a definitive CTX diagnosis in this “cataract cohort” was 3.3% (1/30). Five further patients had moderate elevations of cholestanol levels (10.3, 10.8, 12, 12.4 and 16.5 µmol/L). In the two patients with the highest levels, elevated cholestanol was confirmed once on a subsequent blood sample (16.5 → 13.8 and 12.4 → 15.3 µmol/L). This latter patient was the only one to present a moderately high cholestanol/cholesterol ratio (2.93). Molecular testing found only one heterozygous variant of unknown significance (VUS) in the *CYP27A1* gene (c.1151C > T, p.Pro384Leu), without other exonic or flanking intronic variants, not confirming CTX. The patient was 39 month old at the time of a bilateral cortical cataract diagnosis and cholestanol analysis, with a history of chronic diarrhea but no evidence of neurological disease after extensive diagnostic work-up. Urinary bile alcohols for this patient were tested and found to be normal and the patient has not developed any other sign or symptom of the disease up to last follow up (4 year follow-up). Unfortunately, the remaining four patients with moderate elevations of cholestanol levels, who had normal cholestanol/cholesterol ratios (1.99–2.28) and no personal or family history of neurological disease nor chronic diarrhea, did not undergo *CYP27A1* sequencing. None of the five patients with moderate elevations of cholestanol levels had posterior cataracts, but nuclear (n = 2), cortical (n = 1), anterior (n = 1) and total (n = 1).

286 pediatric patients with a mean age of 14 years (± 4.4 SD, range 1–20 years), 48.3% females, had undergo plasma cholestanol analysis for other reasons than cataract in the same time period in our center (“control” cohort). Although mean cholestanol levels were similar between both “control” and “cataract” cohorts, frequency of elevated cholestanol levels was higher in the “cataract” cohort after excluding the patient with confirmed CTX (5/29, 17.2%) compared to the “control” cohort (12/286, 4.2%) (*p* = 0.003). This was also the case after adjustment for age (*p* = 0.014) (Table 2).

**Table 2:** Patients with juvenile-onset bilateral cataracts versus controls

Demographic characteristics	Patients with juvenile-onset bilateral cataract (n = 29)*	Controls (n = 286)	Significance (*p*)	Significance adjusted for age (*p*)
Female, n (%)	16 (55.2)	138 (48.3)	0.477	
Age at inclusion, y, mean ± SD	6.8 ± 4.3	14.1 ± 4.4	0.001	
Abnormal cholestanol (> 10 µmol/L)	5 (17.2)	12 (4.2)	0.003	0.014
Cholestanol level, µmol/L, mean ± SD	7.3 ± 2.8	6.6 ± 2.2	0.102	

*Y* years

*Excluding the patient with CTX

## Discussion

This is the first real-life prospective cohort studying the value of systematic plasma cholestanol analysis at the moment of juvenile-onset unexplained bilateral cataract diagnosis and before the onset of neurological disease. 1/30 patients (3.3%) was diagnosed with CTX, and had no other symptom of the disease, aside from a personal history of chronic diarrhea in childhood, which can go undetected if not asked directly.

A previous study analyzing plasma cholestanol levels retrospectively in patients with juvenile-onset bilateral cataracts selected from records, reported a prevalence of 1.8% (3/170 patients) [5], and other epidemiological studies in patients with childhood-onset bilateral cataracts have shown a prevalence of 2–3.7% [6–8]. In these studies, cholestanol was analyzed years after cataract diagnosis, once the full CTX phenotype is present and cholestanol levels may not be similar to those at cataract diagnosis. Nevertheless, the rate of CTX diagnosis in patients with bilateral cataracts is high considering the rarity of the disease.

Although descriptions of the cataracts in CTX are largely lacking, four reports also showed posterior subcapsular cataracts with particular fleck-like opacities in patients with CTX [5, 9–11], as was the case in our CTX patient (Fig. 1). The reliability of this particular cataract morphology as a clinical biomarker of CTX should be further studied. Another frequent cause of juvenile posterior cataracts and neurological disease is myotonic dystrophy type 1 (DM1) or Steinert’s disease, however, patients with DM1 frequently show a “Christmas tree” morphology of the cataract [12], which differs from the above mentioned fleck-like opacities in CTX patients (Fig. 1). Inborn errors of metabolism other than CTX causing pediatric cataracts are congenital galactosemia, galactokinase deficiency and Wilson’s disease [13]. In congenital galactosemia, cataract is usually associated with several gastro-intestinal symptoms, and Wilson’s disease frequently associates hepatic manifestations. However, in galactokinase deficiency the cataract may be the only consistent abnormality [14], as it can also be the case in CTX at the moment of cataract diagnosis. Other non-metabolic genetic diseases of bilateral cataracts as well as acquired causes should be searched for [13], guided by a complete ophthalmological and general examination.

As mentioned, chronic diarrhea in CTX can be easily missed during a clinical consultation, as there is no malabsorption or growth retardation and may improve with time. Furthermore, bilateral cataracts can be the sole manifestation of CTX for several years, however, treatment should be initiated as soon as possible. Therefore, even in the absence of chronic diarrhea, neurodevelopmental disorders and fleck-like opacities (which probably increase the likelihood of diagnosing a patient with CTX), patients with juvenile-onset bilateral cataracts should undergo plasma cholestanol analysis.

Patients with bilateral cataracts showed more frequently moderate elevations of cholestanol levels than a control pediatric population (17.2 vs. 4.2%), which are much lower than the levels usually seen in newly diagnosed CTX patients. This could suggest that moderately elevated cholestanol may build up early in the lens and be a risk factor for juvenile cataract development [15]. Age and total cholesterol influence cholestanol levels [16, 17], however in the present study, they do not seem to account for the differing proportion of patients with high cholestanol between cohorts. Nevertheless, differences in sample size may have biased our results and therefore further studies looking specifically for this issue should be undertaken. Whether heterozygous variant in the *CYP27A1* gene may lead to bilateral cataracts without the full CTX phenotype, as reported here for one patient, needs further examination. Alternatively, other genetic variations outside *CYP27A1* gene but in genes involved in bile acids synthesis may account for a moderate increase in plasma cholestanol and be a risk factor for juvenile cataract. Finally, we cannot exclude that among the four non-genotyped patients with moderate elevation of cholestanol, some may have an undiagnosed CTX, although it seems unlikely given the very moderate elevation of plasma cholestanol levels. As this study was part of a real-life clinical scenario, we had some missing data including the lack of global *CYP27A1* genotyping*.* However, the prospective nature of the study reduces selection biases, including recall bias, and has allowed to compile clinical and biochemical data not frequently assessed at the moment of cataract diagnosis.

## Conclusion

Patients with juvenile-onset unexplained bilateral cataract should benefit from a plasma cholestanol analysis for suspected CTX, even in the absence of other CTX clinical manifestations. Whether moderate elevations of plasma cholestanol unrelated to CTX may be a risk factor for bilateral cataracts occurrence needs further examination.

## Data Availability

The datasets used and/or analysed during the current study are available from the corresponding author on reasonable request.
